# Supplementary material for: Why are different estimates of the effective reproductive number so different? A case study on COVID-19 in Germany
Source: PLoS Comput Biol. 2023 Nov 27;19(11):e1011653. doi: 10.1371/journal.pcbi.1011653 (PMC10703420; doi:10.1371/journal.pcbi.1011653)
Supplement: S1 Text — References to relevant code repositories and sources underlying Fig 2, additional remarks on the HZI approach, details on the handling of temporal shifts, additional figures on temporal coherence, extension of the Cori method to a conditional negative binomial distribution. (PDF) [file pcbi.1011653.s001.pdf]

# Supplementary material for Brockhaus et al.: Why are different estimates of the effective reproductive number so different? A case study on COVID-19 in Germany

Contact: E. K. Brockhaus (elisabeth.brockhaus@outlook.de), J. Bracher (johannes.bracher@kit.edu)

## A Repositories from which real-time estimates were obtained

- epiforecasts: https://github.com/epiforecasts/covid-rt-estimates
- ETH: https://github.com/covid-19-Re/dailyRe-Data
- globalrt: https://github.com/crondonm/TrackingR
- HZI: https://gitlab.com/simm/covid19/secir/-/tree/master
- Ilmenau: https://github.com/Stochastik-TU-Ilmenau/COVID-19/tree/gh-pages
- RKI: https://github.com/robert-koch-institut/SARS-CoV-2-Nowcasting_und_-R-Schaetzung, stable version: https://zenodo.org/record/8062328
- rtlive: https://zenodo.org/record/5683308, https://github.com/michaelosthege/rtlive-global
- SDSC: https://renkulab.io/gitlab/covid-19/covid-19-forecast/-/tree/master

## B Sources for generation time distributions shown in Figure 2

We here provide the sources for the generation time distributions used by European public health agencies as displayed in Figure 2.

- Austria: Richter, Schmid and Stadlober: *Methodenbeschreibung für die Schätzung von epidemiologischen Parametern des COVID19 Ausbruchs, Österreich.* https://www.ages.at/fileadmin/Corona/Epidemiologische-Parameter/Methoden_zur_Sch%C3%A4tzung_der_epi_Parameter.pdf. The Cori et al. (2013) method is used for estimation. The generation time distribution was initially set to a gamma distribution with mean 4.46 days and standard deviation 2.63 days. Later this was revised to 3.37 and 1.83 days, respectively.
- Belgium: Sciensano: *COVID-19 Bulletin épidémiologique hebdomadaire (19 mai 2022)*, https://covid-19.sciensano.be/sites/default/files/Covid19/COVID-19_Weekly%20report_20220519%20-%20FR.pdf. The Cori et al. (2013) method is used for estimation. The generation time distribution is set to a gamma distribution with mean 4.7 days and standard deviation 2.9 days (source of parameterization: personal correspondence).
- Czech Republic: Majék et al (2020): *Modelling the first wave of the COVID-19 epidemic in the Czech Republic and the role of government interventions.* medRxiv, https://doi.org/10.1101/2020.09.10.20192070. The generation time distribution is a discrete uniform over $\{4, 5, 6, 7\}$, implying a mean of 5.5 and standard deviation of 1.2.
- Denmark: Statens Seruminstitut: *COVID-19 i Danmark: Epidemiologisk trend og fokus: kontakttal, 11. juni 2020* (2020). https://files.ssi.dk/COVID19-epi-trendogfokus-11062020. The generation time distribution from Nishiura et al. (2020) is used in the Cori et al. (2013) method, which corresponds to a mean of 4.7 and a standard deviation of 2.9 days.
- France: Santé Publique France (2021): *COVID-19 – Point épidémiologique hebdomadaire no 71 du 08 juillet 2021.* https://www.santepubliquefrance.fr/content/download/358653/document_file/COVID19-PE_20210708_signets.pdf. The $R_t$ estimates are obtained using the method by Cori et al. (2013) with a window size of 7 days. The mean and standard deviation of the generation time distribution are not reported, but using trial and error could be reconstructed as approximately 7 and 4.5 days, respectively.

- Italy: Guzzetta and Merler (2020): *Stime della trasmissibilità di SARS-CoV-2 in Italia.* Istituto Superiore di Sanità / EpiCentro. https://www.epicentro.iss.it/coronavirus/open-data/rt.pdf. The method by Cori et al. (2013) is applied; the generation time distribution is a gamma distribution with mean 6.7 days and standard deviation 4.9 days.

- Netherlands: Rijksinstituut voor Volksgezondheid en Milieu (2021): *Covid-19 reproductiegetal.* https://data.rivm.nl/meta/srv/eng/catalog.search;jsessionid=1B3A9B193CB3B1946836BCA3D1BF3A11. The Wallinga-Lipsitch method as implemented in the *EpiEstim* R package (Cori et al., 2020) is used. For pre-Omicron variants, the mean and standard deviation of the generation time are se to 4 and 2 days, respectively. For Omicron, 3.5 and 1.75 days are used (source for standard deviations: personal correspondence).

- Portugal: Instituto Nacional de Saúde (2022): *COVID-19 – curva epidmeica e parâmetros de transmisidade, 18.05.2022.* https://www.insa.min-saude.pt/category/areas-de-atuacao/epidemiologia/covid-19-curva-epidemica-e-parametros-de-transmissibilidade/. The mean and standard deviation of the generation time are set so 3.96 and 4.74 days, respectively (based on Du et al, https://wwwnc.cdc.gov/eid/article/26/6/20-0357_article).

- Scotland: Scottish Government (2020): *Coronavirus (COVID-19): modeling the epidemic in Scotland (Issue No. 24).* https://www.gov.scot/binaries/content/documents/govscot/publications/research-and-analysis/2020/10/coronavirus-covid-19-modelling-epidemic-issue-no-24/documents/coronavirus-covid-19-modelling-epidemic-scotland-issue-no-24/coronavirus-covid-19-modelling-epidemic-scotland-issue-no-24/govscot%3Adocument/coronavirus-covid-19-modelling-epidemic-scotland-issue-no-24.pdf. Estimation is based on the model by Flaxman et al. (2020), which uses a gamma distribution with mean 6.5 and standard deviation 4.11 days (see their Supplementary Information, page 13).

- Sweden: Folkhälsomyndigheten (2022): *Skattning av det momentana reproduktionstalet, 18/05/2022.* Mean and standard deviation of the generation time are set to 4.8 and 2.3 days, respectively. Unfortunately, the respective document is no longer available online.

- Slovenia: Rok Blagus, Manevski and Pohar Perme (2020): *Estimation of the reproductive number and the outbreak size of SARS-CoV-2 in Slovenia.* Slovenian Medical Journal, http://dx.doi.org/10.6016/ZdravVestn.3068. As for Scotland, the model and assumed generation time corresponds to the one from Flaxman et al. (2020).

# C Additional remarks on the HZI approach

## C.1 Determining the generation time distribution

The generation time distribution is not an independent parameter in the SECIR model applied by Khailaie and Mitra et al. (2021) but results from the interplay of several other parameters. The generation time distribution of the model has no closed form. As it arises from the transitions between different compartments in a classic compartmental model, it corresponds to a mixture of convolutions of exponential distributions. For the purposes of our study, we obtain the mean and standard deviation via simulation. Figure A shows compartments of the model, the transitions between which are governed by the following rates and probabilities (see also Knabl, Mitra and Kimpel et al. 2021, particular Supplementary Material p12–13):

- $\alpha = 0.22$, i.e. a 78% probability of entering the state $C_I$ (carrier who will move on to infected) after exposure.

- $R_2 = 1/3.2$ and $R_3 = 1/2$ imply a mean incubation period of 5.2 days.

- $R_4 = 1/7$, i.e., a mean time to recovery of seven days for undetected infected.

- $\mu = 0.085$ is the probability of detection for any infected individual. This value has been taken from the code repository[2] rather than the manuscript.

[2] https://gitlab.com/simm/covid19/secir/-/raw/master/codes/settings/param_random.csv

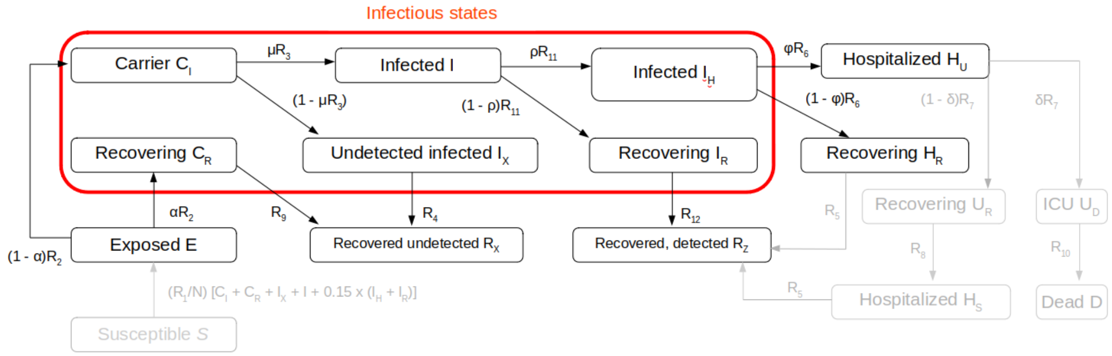


Figure A: Compartmental structure of the SECIR model by HZI. Compartments relevant to the generation time distribution are shown in black, the remaining compartments in grey. This figure reproduces Figure 1 from Khailaie and Mitra et al. (2021).

- $R_{11} = 1/3.7$, i.e., on average it takes 3.7 days to move from $I_H$ to $H_U$. $R_6$ is chosen such that $1/R_6$ and $1/R_{11}$ sum to 4.25 days, which is the assumed time to hospitalization since symptom onset. This implies $R_6 = 1/0.55$.
- $\rho = 0.13$, i.e., the probability that a detected infected patient requires hospitalization (moves to $I_H$) rather than recovering (moving to $I_R$). This value has been taken from the code repository.
- $\varphi = 0.47$, i.e. a hospitalization probability of 47% for individuals reaching the $I_H$ state (but this value is irrelevant for the generation time distribution).
- $R_{12} = 1/3.3$, i.e., the average time left to full recovery for individuals who have already arrived in $I_R$ is 3.3 days.
- The remaining parameters are not relevant for the computation of the generation time distribution and are thus omitted here.

Moreover, Khailaie and Mitra et al. (2021) assume that individuals in the $C_I, C_R, I_X$, and $I$ compartments have the same infectiousness, while infectiousness in the $I_H$ and $I_R$ compartments is reduced by a factor of 0.15. We note that we here only use the assumed mean values of the different parameters and simplifyingly neglect that they are randomly varied around these values in Khailaie and Mitra et al. (2021). To obtain the generation time distribution numerically we then proceed as follows.

1. For a total of 5000 individuals we first sample the path the individual takes through the different compartments from $E$ onwards (e.g., $E \to C_R \to R_X$ or $E \to C_I \to I \to I_H \to H_U$). This involves the probabilities $\alpha, \mu, \rho$ and $\varphi$.
2. We then sample the duration of stay in each of the compartments from exponential distributions with the respective transition rates.
3. For the time spent in infectious compartments $(C_I, C_R, I, I_X, I_H, I_R)$ we sample times of secondary infections from Poisson processes with suitably chosen rates (with diminished intensity for the $I_H$ and $I_R$ compartments). In practice, this is done by first sampling the total number of events from a suitable Poisson distribution and then sampling the respective event times from a uniform distribution over the time spent in the compartment.
4. For each infection event we compute the total time since the entry of the infecting individual in the $E$ compartment. This corresponds to the realized generation time.

We then evaluate the empirical distribution of these generation times. The resulting histogram is shown in Figure B. The mean and standard deviations are given by 10.3 and 7.6, respectively.

Code to reproduce these results is available in https://github.com/ElisabethBrockhaus/Rt_estimate_reconstruction/blob/main/HZI.

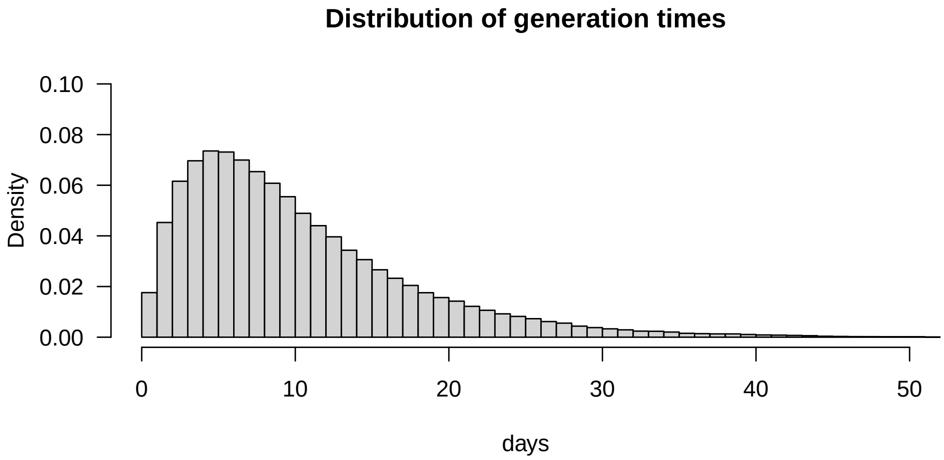


Figure B: Histogram of sampled generation times from the HZI model.

## C.2 Transformation of estimates to standardize generation time distribution

The HZI results proved to be technically challenging to reproduce, and due to the setup of the model, the generation time distribution cannot be manipulated directly in the model. Rather than re-running the model with a different generation time distribution, we, therefore, opted to transform the available estimates to approximate how they would have looked under a different generation time distribution. We employ the following relationship from Wallinga and Lipsitch (2007, Equation 3.6):

$$R = \frac{r}{\sum_{i=1}^{n} y_i\{\exp(-ra_{i-1}) - \exp(-ra_i)\}/(a_i - a_{i-1})}.$$

Here, $R$ is the reproductive number, $r$ is the growth rate, $a_0, a_1, \ldots, a_n$ are the category bounds of a histogram, and $y_1, y_2, \ldots, y_n$ the respective relative frequencies. By plugging in the distribution from Figure B and samples from the consensus distribution Exp(1/4), we can obtain mappings from the growth rate to estimated reproductive numbers under the two generation time distributions (we here use bins of width 0.01 for the histogram). Combining these two, we can map the reproductive numbers computed by HZI under the generation time distribution from Figure B to reproductive numbers under the consensus generation time distribution. This mapping is displayed in Figure C. As one would expect, values of 0 and 1 are mapped to themselves, while otherwise, the values under the consensus distribution (which has a considerably lower mean) are closer to 1.

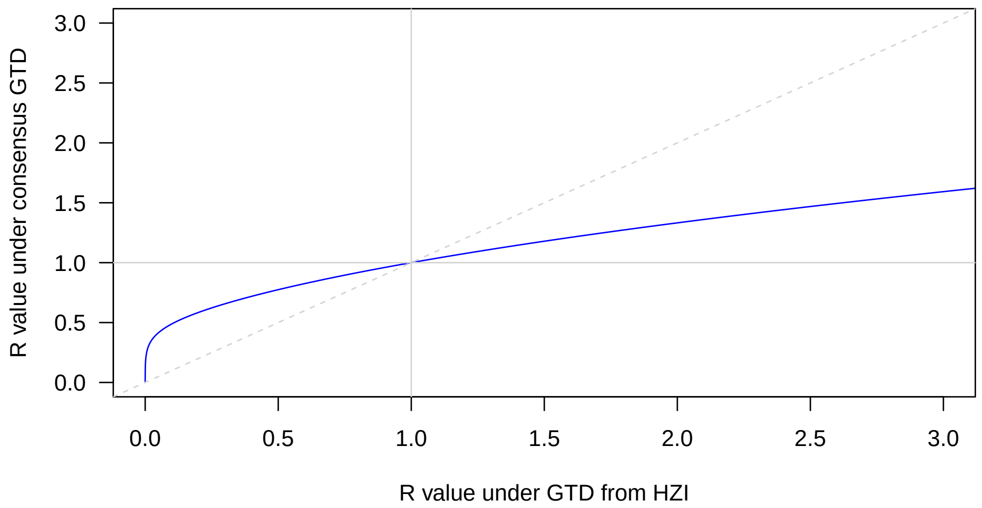


Figure C: Mapping of $R$-values under the generation time distribution used by HZI to R-values under the consensus generation time distribution Exp(1/4).

### C.3 Comparison of estimates with and without additional smoothing

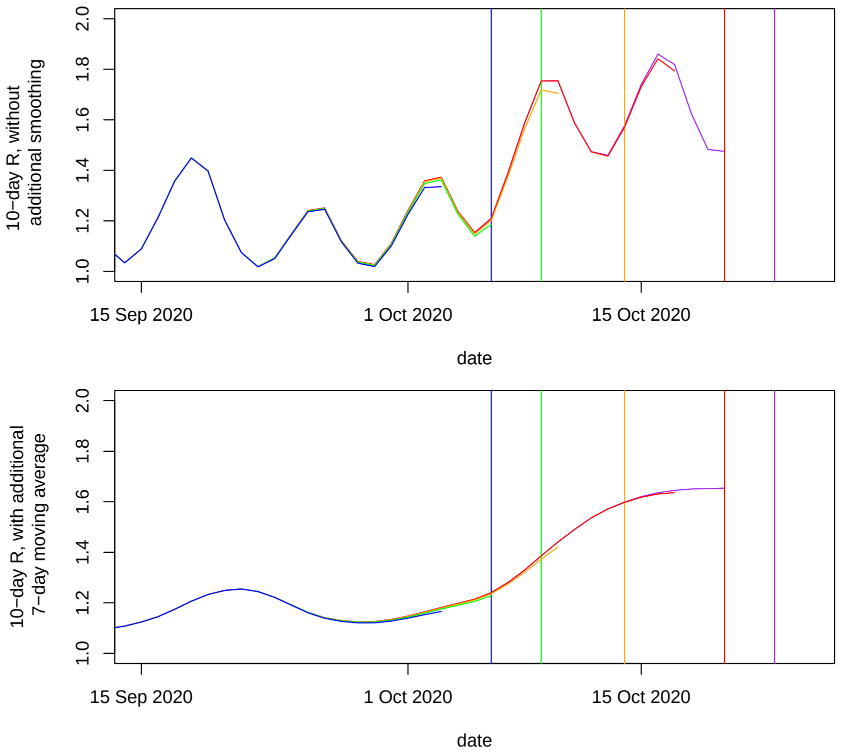


Figure D: Comparison of real-time estimates from the HZI model with and without an additional smoothing step (7-day trailing moving average). Similarly to Figure 4 estimates issued at different dates are overlaid so that retrospective revisions become visible. It can be seen that the additional smoothing step considerably reduces the (typically upwards) retrospective revisions.

# D Details on the handling of temporal shifts

To handle differing assumptions on the incubation period and reporting delay as well as the exact definition of $R_t$ (case vs. instantaneous), we align estimates via simple shifting. We do this in a data-driven way, where for each method we minimize the mean absolute distance to the consensus model. Denoting the days in the considered period by indices $t = 1, \ldots, T$, we thus obtain the shift $s_m$ for each model $m$ as

$$s_m = \text{argmin}_{s \in \{-14, -13, \ldots, 14\}} \sum_{t=1}^{T} |\hat{R}^{m}_{t-s} - \hat{R}^{\text{consensus}}_{t}|.$$

Here we denote by $\hat{R}^m_t$ estimates from model $m$ and by $\hat{R}^{\text{consensus}}_t$ estimates from the consensus model. This approach is based on Alvarez et al. (2021). We note that this is a pragmatic approach and does not minimize the divergence for each pair of methods nor the sum of all divergences.

In Table A, we compare the optimal shifts determined this way by shifts which we can compute from the employed mean incubation periods and reporting delays as provided in the respective manuscripts or code bases. Apart from the HZI model, these agree quite well.

Table A: Shift which minimizes the mean absolute error to the consensus model and explanatory features.

| **Method** | **incubation period** | **reporting delay** | **type of $R_t$** | **shift for $R_t^{\text{case}}$** | **resulting expected shift** | **optimal shift (data-driven)** |
|---|---|---|---|---|---|---|
| ETH | 5.3 (3.2) | 4.4 (3.4) | instant. | 0 | 10 | 10 |
| RKI | 1 | 3.4 (0.4) | instant. | 0 | 4 | 4 |
| Ilmenau | 5 | 2 | instant. | 0 | 7 | 7 |
| SDSC | (sum to 7) | | instant. | 0 | 7 | 7 |
| epiforecasts | 5.4 (2.2) | 5.9 (14.6) | instant. | 0 | 11 | 10 |
| rtlive | 5 | 7.1 (5.9) | case | 4 | 16 | 19 |
| globalrt | 0 | 0 | case | 4 | 4 | 3 |
| HZI | 5.2 | 3.7 | instant. | 0 | 9 | -3 |
| consensus | (sum to 7) | | instant. | 0 | 7 | 7 (fixed) |

.

Step 4a: Shift estimates by the mean of the incubation period and reporting delay distribution.

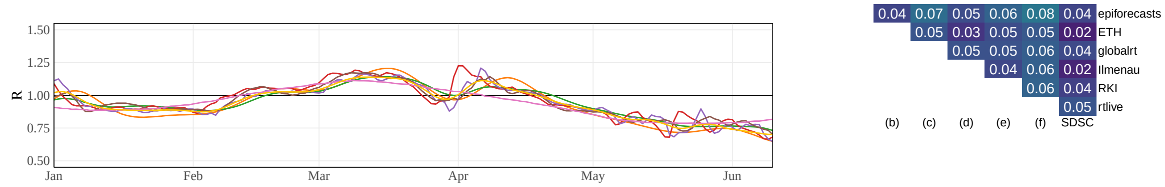


Step 4b: Shift case reproductive number by mean generation time distribution.

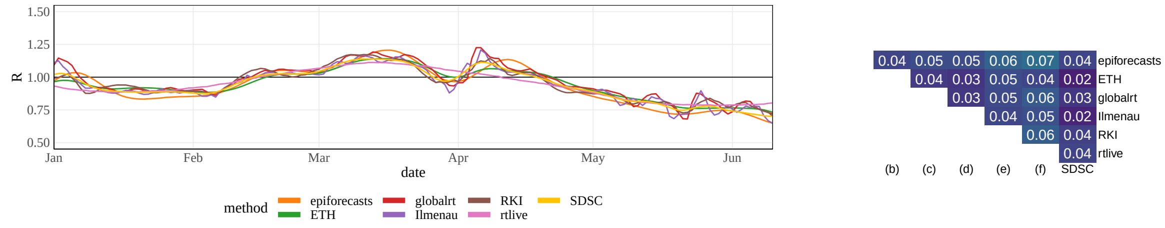


Figure E: Alternative display on the temporal alignment of estimates as in Figure 6, based on information on generation times, incubation periods, and type of $R_t$ from Table A. We split this into two steps and omit HZI as the temporal labeling of estimates obviously does not agree with our reasoning.

# E Supplementary Figures on temporal coherence

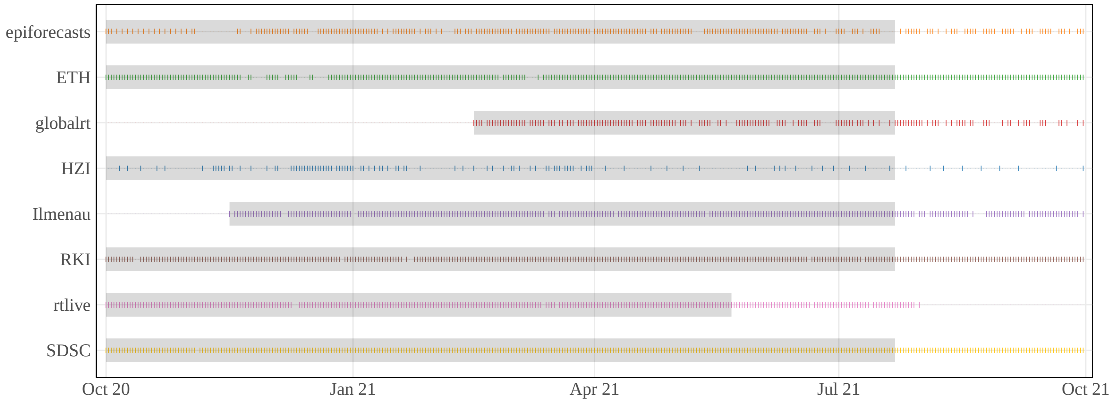


Figure F: Dates for which estimates were published by the research groups. The shaded areas correspond to estimates which are included in the averages in Figure 5. This does not include estimation dates which are only used as consolidated estimates.

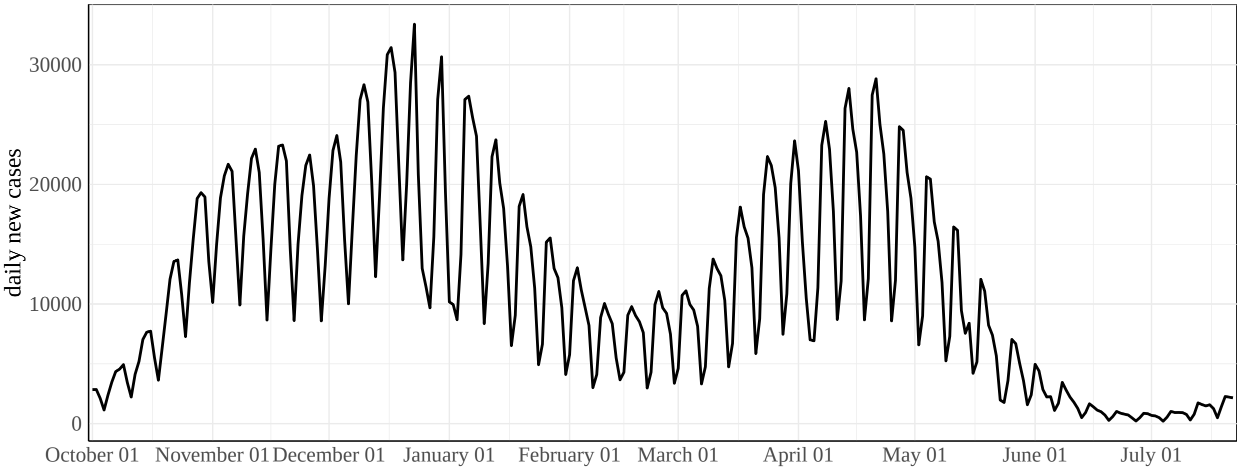


Figure G: Incidence over the time period considered in Section 3 (RKI, positive test).

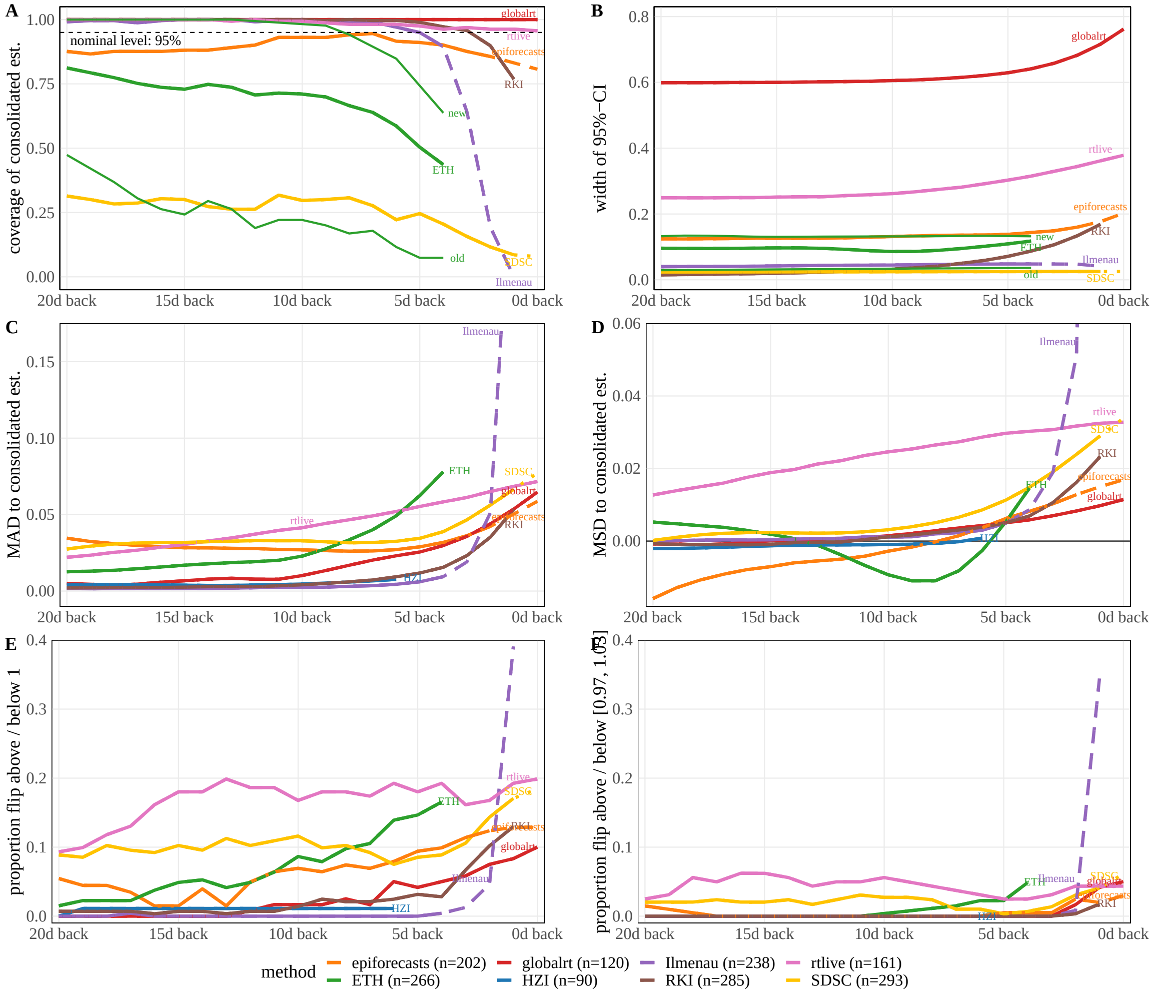


Figure H: Same display as in Figure 5 but with curves shifted by the "optimal shift" from Section 4. Unlike in Figure 5, the horizons are thus approximately aligned, which facilitates comparison. Note that a particularly strong shift results for epiforecasts, where the line segments shown here and in Figure 5 hardly overlap.

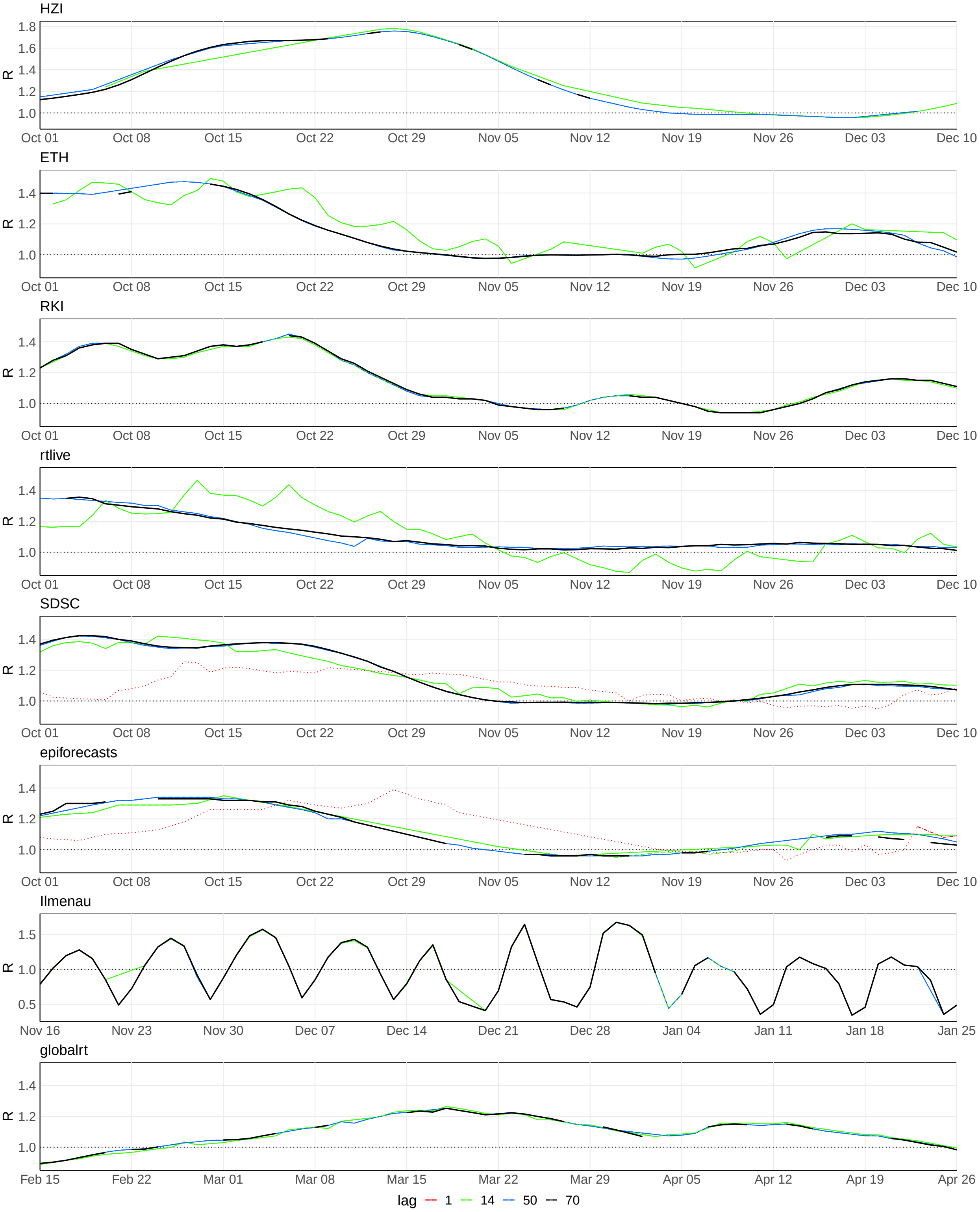


Figure I: Real-time estimates as published 1, 14, 50, and 70 days after the target date.

Step 0: Estimates as published on July 10, 2021 (Figure 1).

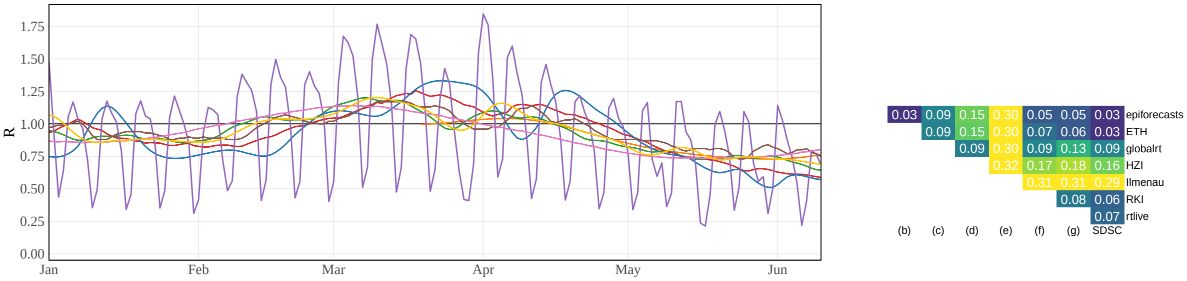


Step 1: Standardize input data to RKI by *Meldedatum*.

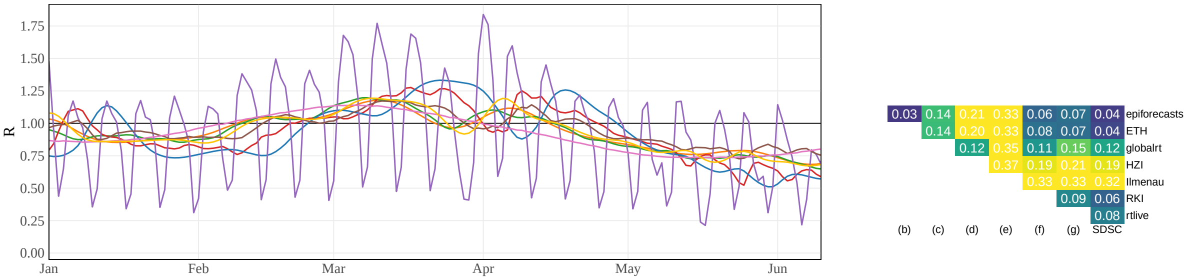


Step 2: Standardize window size in Cori et al. (2013) method to 7 days.

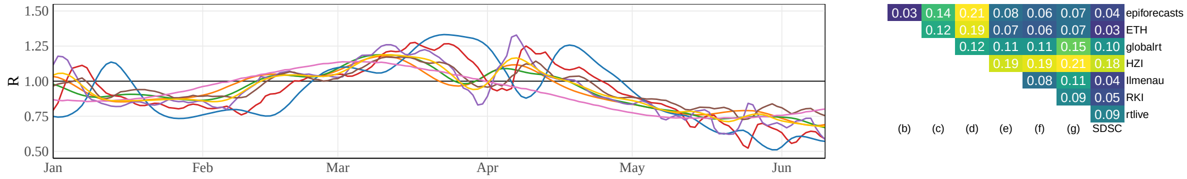


Step 3: Standardize GTD to a gamma distribution with mean 4 and standard deviation 4.

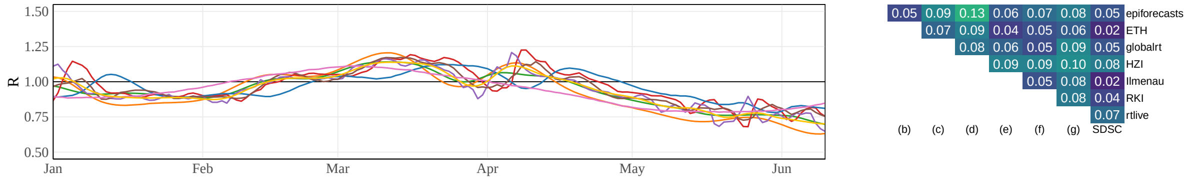


Step 4: Data-driven temporal alignment.

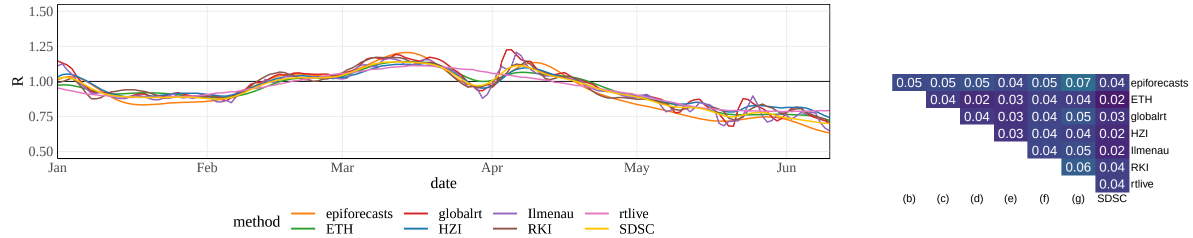


Figure J: Variation of Figure 6: Step-by-step alignment of analytical choices to the consensus specifications. Unlike in Figure 6, the right column shows mean relative rather than absolute differences between point estimates obtained from the different approaches. The relative differences for estimates from models $a$ and $b$ are defined as

$$\text{rel diff} = \frac{|\hat{R}_t^a - \hat{R}_t^b|}{0.5\hat{R}_t^a + 0.5\hat{R}_t^b}.$$

# F Extension of the Cori method to a conditional negative binomial distribution

We provide some details on the extension of the Cori et al. (2013) approach to a conditional negative binomial distribution as used in Section 4.3. As in equation (1) we assume that

$$\mathbb{E}(X_t \mid X_{t-1}, \ldots, X_1) = \lambda_t = R_t^{\text{inst}} \times \sum_{i=1}^{t-1} w_i X_{t-i}. \tag{3}$$

In the classical Cori et al. (2013) approach this is combined with a conditional Poisson assumption

$$X_t \mid \lambda_t \sim \text{Pois}(\lambda_t).$$

Instead, we now use a negative binomial distribution

$$X_t \mid \lambda_t \sim \text{NegBin}(\lambda_t, \psi),$$

which we parameterize by its mean $\lambda_t$ and an overdispersion parameter $\psi$, which we assume to be time-constant. This implies that

$$\text{Var}(X_t \mid X_{t-1}, \ldots, X_1) = \lambda_t + \psi \lambda_t^2.$$

This parameterization is used, e.g., in the endemic-epidemic modeling framework (Held et al. 2007) for infectious disease count time series.

To fit this model to data (specifically, data from a time window of length $w$), we construct a covariate

$$A_t = \sum_{i=1}^{t-1} w_i X_{t-i},$$

such that

$$\mathbb{E}(X_t \mid A_t) = R_t^{\text{inst}} \times A_t.$$

Inference for this negative binomial generalized linear model with an identity link and no intercept can be conducted using the function `glm.nb` from the R package `MASS`:

```
glm.nb(X   -1 + A, link = identity)
```

This estimates the parameters $R_t^{\text{inst}}$ and $\psi$ simultaneously using maximum likelihood inference, providing confidence intervals for both parameters. The practical implementation underlying Figure 9 can be found in the file https://github.com/ElisabethBrockhaus/Rt_estimate_reconstruction/blob/main/otherFiles/epiestim_vs_glm.R in the GitHub repository accompanying this paper.

**Reference:**

Held, L., Höhle, M. and Hofmann M. (2005): A statistical framework for the analysis of multivariate infectious disease surveillance counts. *Statistical Modelling*, 5: 187–199.
