# Supplementary material for: Why are different estimates of the effective reproductive number so different? A case study on COVID-19 in Germany
Source: PLoS Comput Biol. 2023 Nov 27;19(11):e1011653. doi: 10.1371/journal.pcbi.1011653 (PMC10703420; doi:10.1371/journal.pcbi.1011653)
Supplement: Attachment — Submitted filename: Review_Report.pdf [file pcbi.1011653.s002.pdf]

# Review Report

The authors compared different estimates of the effective reproductive number of COVID-19 in Germany. They accessed two types of agreements: within-method agreement and between-method agreement. Their work is interesting because it compared estimates from various perspectives. I have some comments and questions about their work.

1. Figure 4 shows how real-time $R_t$ estimates from different methods evolved over time. The scales and labels of the y-axes are not same for all plots. I would suggest using the same scales and labels.

2. In the panel A of Figure 5, the coverage of Ilmenau achieved close to complete coverage for dates further back in the past, starting from 14 days back. The coverage dropped to 0% at 8 days back. An explanation is necessary for this observation.

3. The author computed mean absolute difference (MAD) in Section 3.2. MAD is not a dimensionless quantity. It would be interesting to compute relative MAD, which quantifies the MAD in comparison to the size of the mean.

4. Figure 5 shows temporal coherence of $R_t$ estimates. As we known, values of $R_t$ above 1 imply epidemic growth, while values below 1 correspond to a decline. I am curious how frequently the real-time and consolidated point estimates are on the same side of 1, i.e., both estimates are larger than 1 or both estimates are smaller than 1.

5. I would suggest using same scales and labels of the y-axes for the $R_t$ plots in Figure 6.

6. In the sequential standardization of analytical choices, the authors did the standardization in the following order: input data, window size, generation time distribution, temporal shifting. The authors should explain the reason for choosing this order.

7. Could the authors provide some insight into what methods we should trust more in what situations?
