# Supplementary material for: Why are different estimates of the effective reproductive number so different? A case study on COVID-19 in Germany
Source: PLoS Comput Biol. 2023 Nov 27;19(11):e1011653. doi: 10.1371/journal.pcbi.1011653 (PMC10703420; doi:10.1371/journal.pcbi.1011653)
Supplement: Attachment — Submitted filename: reply.pdf [file pcbi.1011653.s003.pdf]

Reply to reviewers comments: *Why are different estimates of the effective reproductive number so different? A case study on COVID-19 in Germany* (PCOMPBIOL-D-23-00673)

28th September 2023

We would like to thank the review team for the constructive reports and helpful comments on our paper. In what follows we respond to the different points raised by the reviewers. The page and line references in the referees' statements refer to the original version. We also provide a version of the revised paper with track changes.

Reply to Associate Editor's comments

AE.1: *In particular, include the results for HZI in Figure 5A and B (Reviewer 1), further elaborate the qualitative difference in R_t (e.g. R_t < or > 1) between different methods, provide (if possible) some insights on under what situations or methodological characteristics that would lead to most reliable estimates (Reviewer 2), further consider the generalizability of the study findings (Reviewer 3), such as when there is a much higher variability in R_t for Omicron or other situations, while it is understood that the study didn't adopt a simulation approach which may give an advantage to methods most consistent with the data generating model. In additional to the above comments, please address, 1. Table 2, please avoid abbreviations, or provide the full terms in the footnote.*
Reply: Thank you for the summary and guidance on the main reviewer points, which we will discuss in detail in dedicated sections of this response letter. We followed the additional instructions on abbreviations in Table 2.
Moreover, we are thankful for the clarification regarding the expected scope of the article and potential inclusion of a simulation study. We agree that this extension would be beyond the scope of the paper.

Reply to Referee 1

R1.1: *I think the paper can be interesting to read and it can be helpful for understanding that different models with different assumptions can provide quite different results. Then we may wonder on how to align them and look if they are really incorrect or correct. In my opinion, the analysis was well-conceived and has logic. I recommend it for publication in PLoS CompBio. Some of my remarks, which are not critical, are below. However, my main concern is about keeping the analysis reliable in the "far" future. I am very unsure that many of those github repos listed in the Appendix would stay alive in two or in five years. So it would nice somehow to preserve links to stay working. Probably, by forking the original repos?*
Reply: All real-time estimates used in this paper are available in standardized format the repository `https://github.com/KITmetricslab/reproductive_numbers`, along with information on the licences under which they had been provided by the teams. The codes used to reproduce estimates (relevant for the between-method comparison) are available in the repository `https://github.com/ElisabethBrockhaus/Rt_estimate_reconstruction/` (these are slightly adapted versions of the original codes from the respective teams' repositories). For both repositories we created permanent releases on Zenodo (see code and data availability statement). These two repositories are suffient to reproduce the results from the paper. We moreover encouraged all teams to make permanent releases of their repositories on Zenodo, and have added the respective links to Supplementary Section 1 where available.

R1.2: *the name for Section 2: I wonder why the authors called it "the agony of choice"? In my opinion, it is just about estimating the Rt by different methods. For example, when the meta-analysis performed, people do not usually give their personal assessment for analyzed studies. Here, when the authors called comparing all other studies as being in agony, they unintentionally become subjective.*
Reply: We agree that this heading evokes associations which are not helpful We changed it to *Analytical choices in the estimation of* R_t.

R1.3: *L197: the authors assess the importance of the generation time on estimation of Rt and risk assessment (L190-196), but write nothing about the incubation period. Why exactly do we need IP? Why changing the IP shifts Rt? (L199) This is not clear from the text.*
Reply: Thank you for pointing out this lack of clarity. Like the reporting delay, differences in the incubation period are mainly expected to shift estimates in time. The reason is that the incubation time determines by how much the reconstructed infection events will precede the partly observed disease onset dates or the completely observed reporting dates. In our original formulation this did not become sufficiently clear. We have moved forward the text block previously used for the reporting delay, and refer back to it when discussing the reporting delay:

- "The *incubation period* (IP), i.e., time from infection to symptom onset. Changing the mean incubation time shifts R_t estimates in time (as the

actual infection events will be assumed to precede the respective symptom onsets and reports by a longer or shorter time period). The impact of the variance is not well-studied, but it likely affects the smoothness of estimates.

- The *reporting delay* (RD) between symptom onset and reporting. Like the incubation period, the reposting delay will mainly shift estimates in time."

R1.4: *L139-L155 and further: could the authors indicate what all those acronyms mean? I understood RKI, but what's about ETH, SDSC, Ilmenau, etc.?*
Reply: We added the full names of the respective institutions in brackets.

R1.2: *Figure 5, panel AB: I wonder why I can't see HZI in the plots? If the width of 95% CI is zero, then there should be a line at zero, no?*
Reply: The HZI estimates were publicly displayed without any uncertainty intervals. We interpret this absence as the width of the interval to be unknown (`NA`) rather than zero, and therefore refrained from displaying a line. We now made this more explicit by adding the following to the caption:

> "**B** Mean width of 95%-uncertainty intervals (unavailable for HZI, who only published point estimates)."

If we assumed the 95% uncertainty interval to be of zero width this would imply that the coverage would be 0%, as minimal changes would lead to non-coverage. We think that this would be a misleading summary of the behaviour of the HZI model, the estimates of which are actually among the most stable.

R1.3: *L339: I think it is "Cori et al." method.*
Reply: Thank you for spotting this; we corrected the reference.

R1.4: *L403: Could the authors explain why they write "typically" lags? Does it mean that sometimes it may not be lagged by one generation time period? Could they explain more carefully the lagging issue?*
Reply: We here refer to the following passage from Gostic et al (2020, p7, DOIs added by us):

> "Because the case reproductive number is essentially a convolution of the instantaneous reproductive number and the generation interval (Eq 4) [20], shifting the case reproductive number back in time by the mean generation interval usually provides a good approximation of the instantaneous reproductive number (Sciré et al 2020, `https://doi.org/10.4414/smw.2020.20271`). The case reproductive number generally changes more smoothly than the instantaneous reproductive number (Fraser 2007, `https://doi.org/10.1371/journal.pone.0000758`) (Fig 2B and 2C), but if a smoothing window is used, the estimates become more similar in shape and smoothness."

To avoid confusion we removed the word "typically" as the described pattern holds in general. In Section 2.1, which the sentence in question references, we added the following to explain the lagging issue more conclusively (additions highlighted in bold):

> "We note that R^{case}_{t-i} can be seen as a convolution of R^{inst}_t and the generation time distribution **(Fraser 2007)**. Shifting R^{case}_t back by the mean generation interval m usually leads to good agreement with R^{inst}_t (i.e., R^{case}_{t-m} and R^{inst}_t can be expected to be similar; Gostic et al 2020). **The case reproduction number, however, will change somewhat more gradually; in case of sudden changes discrepancies can thus arise, but these are attenuated if estimation involve smoothing as in most methods considered here (Gostic et al 2020, p.7 and Fig. 2).**"

Reply to Referee 2

R2.1: *The authors compared different estimates of the effective reproductive number of COVID-19 in Germany. They accessed two types of agreements: within-method agreement and between-method agreement. Their work is interesting because it compared estimates from various perspectives. I have some comments and questions about their work.*
Reply: Thank you for your positive and constructive comments.

R2.2: *Figure 4 shows how real-time Rt estimates from different methods evolved over time. The scales and labels of the y-axes are not same for all plots. I would suggest using the same scales and labels.*
Reply: We agree that the results are easier to compare across panels with uniform y-limits and have adjusted the figure accordingly.

R2.3: *In the panel A of Figure 5, the coverage of Ilmenau achieved close to complete coverage for dates further back in the past, starting from 14 days back. The coverage dropped to 0% at 8 days back. An explanation is necessary for this observation.*
Reply: Thank you for pointing out this missing aspect. We added the following (addition in bold) to the interpretation of results in section 3.3:

> "It seems likely that the strong upward corrections in the Ilmenau estimates stem from this aspect as reporting delays were not accounted for explicitly. **We note that data were usually only revised over a few days; afterwards, the Ilmenau estimates thus became quite stable and interval coverage quite high, explaining the characteristic patters in Figure 5.**"

R2.4: *The author computed mean absolute difference (MAD) in Section 3.2. MAD is not a dimensionless quantity. It would be interesting to compute relative MAD, which quantifies the MAD in comparison to the size of the mean.*

Reply: Thank you for this suggestion. We added a version of Figure 6 with mean relative rather than absolute differences as Supplementary Figure 19. Here, we defined the relative differences between the estimates from models a and b as

$$\text{rel diff} = \frac{|\hat{R}_t^a - \hat{R}_t^b|}{0.5\hat{R}_t^a + 0.5\hat{R}_t^b}.$$

To our own surprise the results are practically indistinguishable from the original ones in Figure 6 (though they are not identical, see e.g., Step 2 for the Ilmenau estimates). The reason seems to be that on average R_t is quite close to 1 for all models, without too drastic variation. We nonetheless kept Supplementary Figure S1 and reference it in the main text.:

> "A version of figure 6 with mean relative rather than absolute differences is available in Supplementary Figure S19, but looks similar."

R2.5: *Figure 5 shows temporal coherence of Rt estimates. As we known, values of Rt above 1 imply epidemic growth, while values below 1 correspond to a decline. I am curious how frequently the real-time and consolidated point estimates are on the same side of 1, i.e., both estimates are larger than 1 or both estimates are smaller than 1.*
Reply: Thank you for this suggestion. We included such a display into the figure and added the following description:

> "Panel E shows in which proportion of cases the real-time and consolidated estimates are on opposite sides of the threshold value of 1, i.e., flip between epidemic growth and decline. This is relatively common for recent estimates from Ilmenau, rtlive (more than a third of the cases), and to a lesser degree epiforecasts and SDSC. As can be seen from panel F, these proportions roughly halve if we introduce a tolerance region $[0.97, 1.03]$ and only count instances where the two estimates are on different sides of this region. This implies that many of the flips counted in panel E actually result from minor corrections in cases where $\hat{R}_t \approx 1$. However, some more major revisions remain. We note, though, that even in these cases the uncertainty intervals often contain values on either side of 1, meaning that there is not necessarily a contradiction. For instance, the rtlive intervals very often include values to both sides of 1."

R2.6: *I would suggest using same scales and labels of the y-axes for the Rt plots in Figure 6.*
Reply: Thank you for this suggestion. We tried this display, but unlike for Figure 5 ultimately chose to keep the original version. The reason is that the addition of white space to the bottom three rows obliged us to reduce the sizes of all panels, making the already small plots even more difficult to read. We did, however, adjust the y-axis labelling so labels in all plots are in steps of 0.25. We hope that this will make it more immediately visible that the y-axis limits differ across rows. Also, we point the reader to the differing axis limits in the figure caption:

> "Note that the two top rows we use wider y-axis limits to accommodate the Ilmenau estimates."

A similar note has been added to Figure 7.

R2.7: *6. In the sequential standardization of analytical choices, the authors did the standardization in the following order: input data, window size, generation time distribution, temporal shifting. The authors should explain the reason for choosing this order.*
Reply: The ordering of the standardization is based on the following reasoning:

- We consider the choice of data source somewhat distinct from the other steps, which characterize the statistical / estimation procedure. In our opinion, the compounding of these steps is the main interest of Figure 6, while the impact of the data source is well-described by Figure 7. It thus seems natural to standardize the data source first, thus removing its impact before directing attention to the other dimensions.
- The choice of window size has a very strong impact on results (in particular for Ilmenau). The roles of the generation time and temporal shift would be difficult to discern without standardizing the window size first. We therefore chose to assign it to Step 2.
- The temporal shifting step is handled in a data-driven way, which requires the other aspects to have been aligned first. We therefore handle this aspect in Step 4.
- This leaves Step 3 for the generation time distribution.

We now provide an abridged version of this reasoning in the text:

> "The order of steps is motivated as follows. It seems natural to handle the data source first (Step 1), establishing a common basis for the remaining aspects. As the choice of window size has a strong impact, we handle it second (Step 2), thus avoiding that it obscures the picture in the following steps. As the temporal shift is handled in a data-driven way, it needs to be handled last (Step 4), leaving the generation time distribution to be handled in Step 3."

R2.8: *Could the authors provide some insight into what methods we should trust more in what situations?*

Reply: Our analysis does not permit us to declare one or two methods the most trustworthy ones. This is because we are unable to assess which methods came closest to the true R_t values; however, there are a number of learnings which can guide a reader in choosing among a multitude of available estimates.

- Our study shows that the impact of the epidemiological parameterization on estimates is at least as strong as that of the statistical method. We therefore argue that successful R_t estimation is based on careful parameterization and transparent communication thereof. An aspect which could have received more attention in this respect is the updating of parameterizations in the light of new evidence or changing virus properties. As an example, the generation time distribution used by the Dutch public health authorities was updated for the omicron variant. Among the estimation schemes considered in our paper, such an update was only done by ETH, but after the considered time period. As a matter of fact, many of the employed parametrizations stem from the very early phases of the pandemic and are based on rather small data sets. In presence of multiple R_t estimates we thus recommend to carefully assess how justified and up-to-date their respective parameterizations are. We added a short paragraph on this aspect to the discussion.

  > "Given the important role of these parameterizations, we recommend to assess their plausibility carefully when selecting among different sets of R_t estimates. The parameterizations should be backed by recent and solid evidence from the literature. In practice, many R_t estimates for COVID-19 continued to be based on rather uncertain evidence from the early phases of the pandemic, without updates in the light of new evidence or virus properties. The ETH team updated their generation time distribution to a new estimate for the Omicron variant (Manica et al 2022) retrospectively in October 2022; a similar change was made e.g., by the Dutch National Institute for Public Health and the Environment (RIVM). Such revisions may be helpful to ensure methods stay up-to-date."

- At least for retrospective estimation we found that rather simple methods can yield point estimates which are quite similar to more sophisticated methods, provided comparable parameterization. However, the corresponding uncertainty intervals may be too narrow as they reflect too few sources of uncertainty. We consider this a relevant difficulty in the application of the Cori et al method. In the discussion we now write:

  > "In terms of the uncertainty intervals of retrospective estimates we found a certain divide between approaches based on the method by Cori et al (2013) and more sophisticated Bayesian approaches, which yielded wider uncertainty intervals. As discussed in Section 4.3 the former may tend

> to underestimate the overall estimation uncertainty, which arises from the compounding of various factors.”

- Some methods make more effort than others in reconstructing the time series of infections, putting in place sophisticated deconvolution schemes. This level of detail may lead to better estimation in times of rapid change, when careful deconvolution and simpler smoothing and shifting lead to different results. In times of stability or gradual change, this aspect may be less decisive. In our study, however, we could not assess whether more sophisticated estimates are actually more accurate in times of rapid change; this aspect can only be studied in simulation experiments. In the discussion we now write:

  > “We expect that careful deconvolution of incidence data may yield a clearer picture than simpler shifting or smoothing approaches especially when R_t changes abruptly; this, however, would need to be assessed in simulation studies where the true R_t values are known.”

- If estimates are to be used in real time, as is often the case, it needs to be determined up to how many days before the publication date a method can provide temporally coherent estimates and uncertainty intervals. Methods which can provide such estimates for more recent dates are generally preferable. Here, however, it should be taken into account how the estimates are assigned to different dates.

As a more general rule, we recommend to keep an eye on a few different sets of estimates. This way one can spot cases where an estimate deviates substantially or systematically from those of other methods, and dig deeper to understand why this is the case. As already discussed in the manuscript, we moreover advise to monitor estimates based not only on different methods and assumptions, but also different data sources (Sherratt et al 2022). We expect these to yield more independent pieces of information, contributing to a thorough situational assessment.

Reply to Referee 3

R3.1*: In this study, the authors investigated why different estimates of estimates of the effective reproductive number are so different. The article presents an interesting perspective, but there are still some issues that need to be addressed urgently.*
Reply: Thank you for your positive and constructive comments.

R3.2*: The authors used Germany as an example and found that many parameters will ultimately affect the estimation results. However, using only one example seems somewhat insufficient. We are very curious as to whether these results can be validated using simple and controllable simulation data. Modifying parameters (data source, data pre-processing, assumed generation time distribution, statistical tuning parameters and various delay distributions) to generate simulated data and then using these methods for estimation can help evaluate within-method temporal coherence and between-method agreement of retrospective estimates.*

Reply: Thank you for this suggestion, which we discussed extensively within the authorship team. We ultimately reached the decision not to include such a simulation study in our paper. The main reasons are as follows.

- **Scope of the paper**: as argued in the introduction (lines 78–91), our paper has been conceived as complementary to existing studies taking a forecasting or simulation angle. Examples of the latter include the extensive works by Gostic et al and O'Driscoll et al. Further work along these lines has been published e.g., by Annunziato and Asikainen (2020, EU Joint Reserach Centre, `https://doi.org/10.2760/036156`) and Teh et al (2022, JRSSA, `https://doi.org/10.1111/rssa.12971`). While we agree that there could be interesting additions to the simulation-based literature based on our framework, we consider them out of the scope of the present paper.
- **Conceptual difficulties:** As mentioned in the manuscript, the achilles heel of many R_t simulation studies is that they essentially measure proximity between the assumptions formulated for the data-generating process and those made by the compared statistical methods. Any simulation study run under the assumptions a particular group of authors deemed reasonable will favour their approach over others. It is unclear to us how a meaningful and fair choice can be made here.
- **Length of the paper:** The manuscript is already longer than the typical PLOS Computational Biology publication and we think that adding even more material may deter rather than attract readers.

This decision is in line with the editorial assessment that

> "[...] it is understood that the study didn't adopt a simulation approach which may give an advantage to methods most consistent with the data generating model."

We hope for the reviewer's understanding concerning this decision.

R3.3: *Page 5. The authors should summarize these methods in a table, listing the requirements for input data, the form of output results, and the calculation principles, etc.*
Reply: Thank you for this suggestion. We have extended Table 2 in order to cover the aspects raised by the reviewer. We have added rows on the programming language, further data inputs (other than case incidence data) and output format.

R3.4: *The authors should organize the results in a clear and concise manner for better understanding, rather than presenting them in a large block of text, such as section 4.2.*
Reply: Thank you for this comment. Re-reading the manuscript with some temporal distance we agree that the unstructured text blocks are difficult to digest. To better guide the reader we now use bold paragraph headings to give sections 3.1, 3.2, 3.3, 4.1 4.2 and 4.3. some internal structure.

R3.4: *The authors should explain what is meant by a "consolidated point" and further elaborate on the important role of the four indicators.*
Reply: Thank you for pointing this out. We agree that the notion of the consolidated estimate was not clearly introduced at its first occurrence. We added the following to the introduction of Section 3 (addition in bold):

> "Our analyses are based on real-time estimates obtained from the repositories referenced in Supplement S1. **These will be compared to *consolidated* estimates issued at a later time point by the same method, using data which can be assumed to have stabilized; see the following subsections for details.**"

As the analyses presented in Sections 3.1 and 3.2 require consolidated values with slightly different definitions, we made both of them more explicit. In Section 3.1 we now write (addition in bold):

> "For each method and a 70-day period, it overlays real-time estimates and a **consolidated** estimate made six months later (black line) when all data and results can be expected to have stabilized."

In Section 3.2 we extended the description as follows:

> "To substantiate these observations, we assess the temporal coherence of estimates quantitatively. Unlike **in the illustration** in Figure 4 we do not use estimates made at a single later time point as the consolidated ones. Instead, **for each target date the consolidated estimate is defined as the estimate generated 70 days later by the same method.** This ensures that the time during which the estimates could be revised is the same for all target dates. Based on this definition we computed the following."

Concerning the motivation of the considered indicators we extended their description in Section 3.2 as follows (additions in bold):

- "The fraction of instances in which the 95% uncertainty intervals issued in real-time covered the respective consolidated point estimate. **If this quantity falls substantially below the nominal level of 0.95, there is an indication that the estimates are temporally incoherent.**
- The average width of 95% uncertainty intervals. **This serves to contextualize the coverage fractions, which depend on both the degree of revisions and the width of uncertainty intervals.**
- The mean absolute difference (MAD) between real-time and consolidated point estimates. This reflects the volatility of real-time estimates relative to the consolidated ones**, thus summarizing their temporal stability.**

- The mean signed difference (MSD) of real-time and consolidated estimates. This reflects if revisions are systematically in one direction. We orient this such that positive values indicate upwards corrections.
- **The fraction of instances in which the point estimate flipped from $\hat{R}_t < 1$ to $\hat{R}_t > 1$ or vice versa. This describes how commonly the qualitative interpretation as epidemic growth or decline changes. Note that this is based purely on the point estimate and ignores the uncertainty intervals.**
- **The fraction of instances in which the point estimate flipped from $\hat{R}_t < 0.97$ to $\hat{R}_t > 1.03$ or from $\hat{R}_t > 1.03$ to $\hat{R}_t < 0.97$. Instances where an initial estimate close to one crossed this threshold via a minor revision are thus omitted, meaning that we focus on more major revisions.**"

Note that the two last points have been added in response to comment 2.5 from Reviewer 2.

R3.5: *Figures 6 and 7 are difficult to understand. Besides the consistency among the methods, what does the mean absolute differences between point estimates represent?*
Reply: The mean absolute differences indeed serve to illustrate the consistency among methods, thus supporting the visual impression gained from the left column. The display via heatmaps serves to provide a more easily perceivable visual representation, allowing the reader to spot easily where major differences remain. At the request of Referee 2, comment 2.4, we now also provide a version of this plot with relative rather than absolute differences.
We think that the mean absolute differences are a meaningful summary measure as they are on the same scale as the estimates themselves and can be related to their realistic range. As pointed out in Section 4.2, the estimates studied in our analysis are mainly between 0.75 and 1.25. We thus consider the average pairwise mean absolute difference of 0.15 encountered in the original estimates quite substantial. After standardizing all aspects other than the estimation method, the mean absolute differences drop to the order 0.02–0.05 for most pairs of methods, which we consider a rather minor difference.

Other changes

- By now all considered estimation pipelines have been retired. We added the respective end dates to Table 1.
